# Water Deficit and Seasonality Study on Essential Oil Constituents of *Lippia gracilis* Schauer Germplasm

**DOI:** 10.1155/2014/314626

**Published:** 2014-09-11

**Authors:** Elizangela Mércia de Oliveira Cruz, Jéssika Andreza Oliveira Pinto, Saymo Santos Fontes, Maria de Fátima Arrigoni-Blank, Leandro Bacci, Hugo César Ramos de Jesus, Darlisson de Alexandria Santos, Péricles Barreto Alves, Arie Fitzgerald Blank

**Affiliations:** ^1^Department of Agronomic Engineering, Federal University of Sergipe, Avenue Marechal Rondon s/n, 49100-000 São Cristóvão, SE, Brazil; ^2^Department of Chemistry, Federal University of Sergipe, Avenue Marechal Rondon s/n, 49100-000 São Cristóvão, SE, Brazil

## Abstract

The aim of this study was to analyze the chemical composition of the essential oil from leaves of *Lippia gracilis* genotypes, in the dry and rainy seasons, and with and without irrigation. The extraction of essential oil was realized by hydrodistillation in a Clevenger apparatus. The chemical composition analysis was performed using a GC-MS/FID. The leaves of the *L. gracilis* genotypes provide essential oil with content between 1.25% and 1.92% in the rainy season and 1.42% and 2.70% in the dry season; when irrigation was used the content was between 1.42% and 2.87%, without irrigation contents were between 1.60% and 3.00%. The chemical composition of *L. gracilis* showed high levels of terpenes. The major constituent of genotypes LGRA-106 was thymol and carvacrol was the major constituent for the other genotypes. Concentrations showed little variation between seasons, demonstrating the stability of the chemical composition of *L. gracilis* even with different climatic conditions.

## 1. Introduction

The genus* Lippia* contains about 200 species of aromatic plants, which can be herbaceous, shrubs, and even small trees. Most of the species are native to America and Africa, grow in sandy soils along rivers and lakes, in regions with tropical and subtropical climate [1]. In Brazil, the genus is represented by 120 species characterized by its strong and pleasant fragrance [2].

Some species of the genus* Lippia* are characterized by the presence of essential oils with antimicrobial activity due to the presence of the phenolic monoterpenes, thymol and carvacrol. Among these species,* Lippia gracilis* Schauer (Verbenaceae), native to northeastern Brazil, has been highlighted by presenting high levels of these monoterpenes [3].

*L. gracilis* is a deciduous, branched shrub, up to 2 m in height, proper of the northeast semiarid vegetation of well drained lands [4]. The aromatic leaves, together with the flowers, constitute the medicinal part of the plant, from where essential oil is extracted [5, 6]. The major components found in* L. gracilis* are very varied, such as carvacrol, p-cymene, *γ*-terpinene, *β*-caryophyllene, and thymol. [2].

The chemical composition of secondary metabolites is related to three factors: genetic, environment, and cultivation techniques. Within the climatic parameters, atmospheric temperature and rainfall have been identified as factors that influence the composition and content of essential oil in aromatic plants [7].

The composition of the essential oil of a plant is genetically determined and is usually specific to a particular organ and characteristic for the stage of development [8], but environmental conditions are capable of causing significant variations, giving origin to chemical diversity in plants rich in essential oils [9]. Differences in the chemical composition of essential oils are not only a product of the influence of environmental factors, but also reflect the genotypic variation of these plants [10].

So, we observe that there is a very large difference in yield and chemical composition of essential oils of species in different environments, caused by differences in productive efficiency of active compounds. Still, it should be noted that the time you get higher essential oil production may not be the time of greatest production of the chemical constituent of interest [11].

The aim of this work was to evaluate the effect of harvesting time and the availability of water for the plant on the chemical composition of the essential oils of* L. gracilis* genotypes.

## 2. Materials and Methods

### 2.1. Plant Materials

The genotypes of* L. gracilis* (Table 1) used were obtained from collections realized in the States of Sergipe and Bahia, recorded and identified in the ASE Herbarium of the Federal University of Sergipe. Cuttings collected from a single plant per genotype were used for preparing seedlings that were used to implement the assay.

### 2.2. Influence of Harvesting Seasons and Irrigation on* L. gracilis *Genotypes

The experiments were conducted at the “Campus Rural da UFS” Research Farm, located in São Cristóvão, Sergipe State, Brazil, from 2009 to 2010. The climate of the region is tropical semiarid, and the soil is classified as Red-Yellow Argisol. A randomized block design was used for the experiments, with three replicates in a split-plot in time design.

For both experiments fertilizer was applied 15 days before transplantation of the plants to the field. Each plot consisted of four rows of four plants, and the four centered plants were harvested to obtain the data. Fertilizing was realized 15 days before transplantation of the plants to the field, using 5 liters of cattle manure per plant. After the harvest of the rainy season plants were fertilized with 3 liters of cattle manure per plant. Spacing used in this experiment was 1.0 × 1.0 m. When necessary, weeding was performed manually.

**Figure 1:**
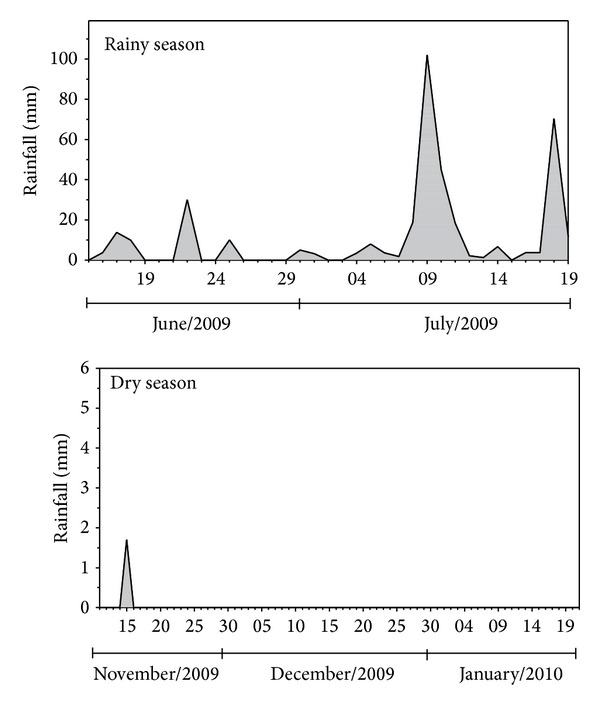
Rainfall (mm) in the experimental area in the rainy and dry seasons.

In the plots of the first experiment, seven genotypes of* L. gracilis* (LGRA106, LGRA107, LGRA108, LGRA109, LGRA110, LGRA201, and LGRA202) were tested. In the subplots, two harvest seasons (rainy and dry seasons) were tested. The harvests of leaves to obtain the essential oil were performed in July 2009 (rainy season) and December 2009 (dry season). At each harvest the plants were cut and the fresh weight was measured. The leaf removal of the harvested plants was done manually and drying was done in an oven with forced air circulation at 40°C for five days. Rainfall data were collected during the conduction of the experiment (Figure 1).

In the plots of the second experiment, the same seven genotypes of* L. gracilis* used in the first experiment were tested. In the subplots, two irrigation systems (with and without irrigation) were tested. The subplot with irrigation consisted of a daily drip irrigation, applying 6 mm*·*day^−1^.

The experiment was established in January 2009 and the harvest was realized on January 24, 2010. In the period of November 16, 2009 to January 24, 2010, there was no rainfall in the experimental area region.

### 2.3. Essential Oils Distillation

The essential oils of dry leaves (samples of 75 g) were obtained by steam distillation using a Clevenger apparatus for 140 min [12]. The content percentage was expressed in % (mL per 100 g of dry leaves). Essential oil yield was calculated with the following formula:

(1) Yield  in  mL·plant−1 =essential  oil  content  (%)100  ·weight  of  dry  leaves  per  plant  (g).

### 2.4. Analysis of Essential Oils

The analysis of the essential oil chemical composition was performed in a gas chromatograph coupled to a mass spectrometer (GC-MS) (Shimadzu, model QP 5050A) equipped with an AOC-20i auto injector (Shimadzu) and a fused-silica capillary column (5%-phenyl-95%-dimethylpolysiloxane, 30 m × 0.25 mm id., 0.25 *μ*m film, J&W Scientific). Helium was used as the carrier gas at a flow rate of 1.2 mL/min. The temperature program was as follows: 50°C for 1.5 min, temperature increase at 4°C/min until reaching 200°C, and temperature increase at 15°C/min until reaching 250°C and 250°C for 5 min. The injector temperature was 250°C and the detector (or interface) temperature was 280°C. The injection volume of ethyl acetate was 0.5 *μ*L, the partition rate of the injected volume was 1 : 87, and the column pressure was 64.20 kPa. The mass spectrometer conditions were as follows: ionic capture detector impact energy of 70 eV and scanning speed 0.85 scan/s from 40 to 550 Da.

Quantitative analysis of the chemical constituents was performed by flame ionization gas chromatography (FID), using a Shimadzu GC-17A (Shimadzu Corporation, Kyoto, Japan) instrument, under the following operational conditions: capillary ZB-5MS column (5% phenyl-arylene-95%-dimethylpolysiloxane) fused silica capillary column (30 m × 0.25 mm i.d. × 0.25 *μ*m film thickness) from Phenomenex (Torrance, CA, USA), under same conditions as reported for the GC-MS. Quantification of each constituent was estimated by area normalization (%). Compound concentrations were calculated from the GC peak areas and they were arranged in order of GC elution.

The essential oil components were identified by comparing their mass spectra with the available spectra in the equipment database (NIST05 and WILEY8). Additionally, the measured retention indices were compared with those in the literature [13]. The relative retention indices (RRI) were determined using the Vandendool and Kratz [14] equation and a homologous series of* n*-alkanes (C_8_–C_18_) injected under the chromatography conditions are described above.

### 2.5. Statistical Analysis

We analyzed the following variables: essential oil content (%) and yield (mL*·*plant^−1^). The content (%) of the following chemical constituents were determined: α-thujene, myrcene, α-terpinene, p-cymene, limonene, 1,8-cineol, *γ*-terpinene, linalool, methyl thymol, thymol, carvacrol, *β*-caryophyllene, α-Humulene, bicyclogermacrene, spathulenol, and caryophyllene oxide.

The means of the variables were subjected to the analysis of variance *F* test and were compared using the Scott-Knott test at 5% probability. The multivariate statistical analysis of main components and Euclidean distances were realized with the Statistica 7.0 software.

## 3. Results and Discussion

### 3.1. Influence of Harvesting Seasons on* L. gracilis *Genotypes

The leaves provided yellowish essential oils with an average content of 1.55% in the rainy season and 2.09% in the dry season. The LGRA201 and LGRA202 genotypes had higher yields and contents, independent of the harvest season; in the dry season there was only a significant difference for LGRA106 and LGRA109, and in the rainy season no significant difference occurred in both the yield and content.

Sixteen compounds were identified in the essential oil of* L. gracilis* genotypes, where seven compounds had higher concentrations than 2%, being thymol the major compound in LGRA106 genotype (average of 58%), with low contents of carvacrol (Table 2). In the other genotypes carvacrol was the major compound (average of 40%), and the contents of thymol were low (Table 2). The contents of thymol ranged from 3.25% (LGRA108) in the rainy season to 6.95% (LGRA201) in the dry season. Similar results were found by [2] whose components are mostly terpenes and for all samples the major component was the same with small changes.

The thymol/carvacrol inversion can cause different responses when the essential oil is used in biological tests, since thymol is used to treat infections of mouth, throat, and skin, and carvacrol has potent anti-inflammatory and antimicrobial activity [15]. In the case reported by [16], there is interaction between carvacrol and thymol, resulting in a synergistic action between them, enhancing the activity of both for control of bacterial cells.

Concentrations showed little variation between seasons, with significant differences only in genotypes LGRA106 for thymol; LGRA108 and LGRA202 for carvacrol; LGRA107 and LGRA109 for *β*-caryophyllene. This demonstrates the stability of the chemical composition of the essential oil of* L. gracilis*, even with different environment conditions. A small variation also was observed in the chemical composition of the essential oils of basil in 2005 and 2006 [17].

The percentages of myrcene, 1,8-cineole, and *γ*-terpinene also varied little in the different seasons, where the dry season showed the highest percentage, with the exception of myrcene whose percentages were higher in the rainy season. Myrcene ranged from 2.05 (LGRA108) to 3.20% (LGRA201) in the rainy season, and from 1.8% (LGRA108) to 2.74% (LGRA201) in the dry season. The *γ*-terpinene and methyl thymol exhibit wide variation among genotypes and seasons with percentages varying from 3.50% (LGRA106) in the dry season, to 21.11% (LGRA201) in the rainy season and from 0.19% (LGRA201) in the rainy season, to 10.78% (LGRA106) in the dry season, respectively. The genotype LGRA109 does not contain 1,8-cineole; the absence of this constituent may be related to plant metabolism [18].

The results of this study indicate some stability in the composition of the essential oil of* L. gracilis* in different environmental conditions. Divergent results were found in genotypes of* L. sidoides* harvested at different seasons [19].

**Figure 2:**
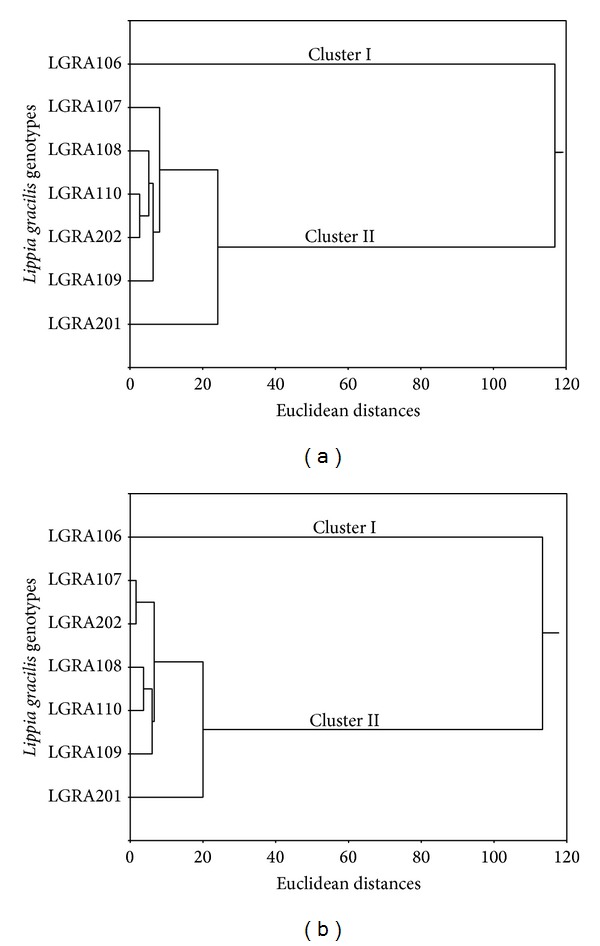
Bidimensional dendrograms representing the similarity of the chemical composition between seven* L. gracilis* genotypes for plants harvested in the rainy (a) and dry (b) seasons.

Considering the similarities of the chemical constituents of the essential oil of the seven genotypes, we note that two distinct clusters were formed, independent of the season (Figure 2). The clusters were characterized as Cluster 1: genotype LGRA106, whose major constituent is thymol; Cluster 2: with the other genotypes (LGRA107, LGRA108, LGRA109, LGRA110, LGRA201, and LGRA202), which present carvacrol as major compound (Figures 2(a) and 2(b)).

**Figure 3:**
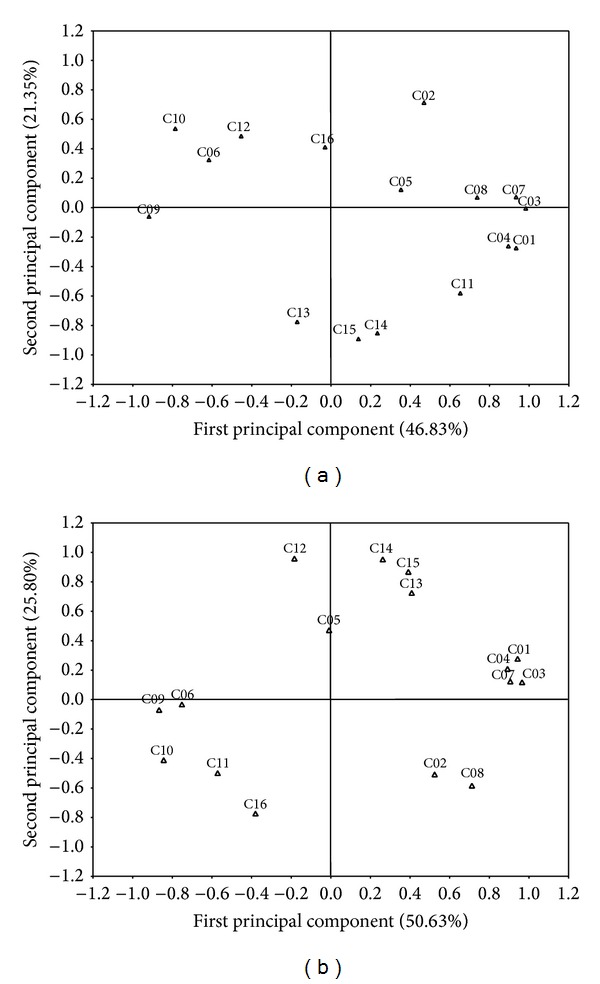
Distribution of the chemical constituents of the essential oil of* L. gracilis* in relation to the two principal components through analysis of the principal component analysis (PCA) for plants harvested in the rainy (a) and dry (b) seasons. (C01 = α-thujene, C02 = myrcene, C03 = α-terpinene, C04 = p-cymene, C05 = limonene, C06 = 1,8-cineol, C07 = *γ*-terpinene, C08 = linalool, C09 = methyl thymol, C10 = thymol, C11 = carvacrol, C12 = *β*-caryophyllene, C13 = α-humulene, C14 = bicyclogermacrene, C15 = spathulenol, and C16 = caryophyllene oxide).

According to principal component analysis (PCA) (Figures 3(a) and 3(b)), the first principal component represented 46.83% and 50.63% of the total variance for the rainy and dry season, respectively. The second principal component represented 21.35% and 25.80% of the total variance for the rainy and dry season, respectively (Figures 3(a) and 3(b)).

The first principal component related positively to α-thujene (*r* = 0.93), α-terpinene (*r* = 0.98), p-cymene (*r* = 0.89), and *γ*-terpinene (*r* = 0.93) and negatively to methyl thymol (*r* = −0.91) and thymol (*r* = −0.79), in the rainy season. In the dry season the first principal component related positively to α-thujene (*r* = 0.94), α-terpinene (*r* = 0.96), p-cymene (*r* = 0.89), and *γ*-terpinene (*r* = 0.90) and negatively to methyl thymol (*r* = −0.87) and thymol (*r* = −0.84) (Figures 3(a) and 3(b)).

The second principal component related positively to myrcene (*r* = 0.71) and negatively to bicyclogermacrene (*r* = −0.85) and spathulenol (*r* = −0.89), in the rainy season. In the dry season the second principal component related positively to *β*-caryophyllene (*r* = 0.96), bicyclogermacrene (*r* = 0.95), and spathulenol (*r* = 0.87) and negatively to caryophyllene oxide (*r* = −0.77) (Figures 3(a) and 3(b)).

These results confirm the existence of two chemotypes of* L. gracilis*, one having thymol as principal marker and the other carvacrol, similarly to [20] which were able to determine the presence of different chemotypes in* L. graveolens* by the multivariate analysis of the essential oil constituents.

### 3.2. Influence of Irrigation on* L. gracilis* Genotypes

This assay showed essential oil with 16 compounds, with a high percentage of monoterpenes, regardless of the irrigation. According to the average test (Table 3), the genotypes LGRA201 and LGRA202 obtained the highest averages for essential oil content and yield, when compared to the other genotypes.

Comparing the treatments with and without irrigation we observed that the values of essential oil content and yield of all genotypes are statistically equal. Very low values of essential oil content in irrigated plants of* Piper hispidum* also were observed in [21]. In* L. sidoides* it was observed that genotypes harvested in the dry season showed higher content and yield of essential oil [19].

Compared to the averages of the main chemical compounds, the treatments with and without irrigation had varied effect, since some averages were higher with irrigation, and other without irrigation. The percentage of thymol, which is the major compound of genotype LGRA106, was higher in the treatment with irrigation, since carvacrol, which is the major compound of LGRA109, obtained higher mean in the treatment without irrigation (Table 3).

The essential oil of the genotypes LGRA107, LGRA108, LGRA109, LGRA110, LGRA201, and LGRA202, presented carvacrol in higher percentage. The chemical composition of the essential oil in the different genotypes showed little changes between the treatments with and without irrigation.

**Figure 4:**
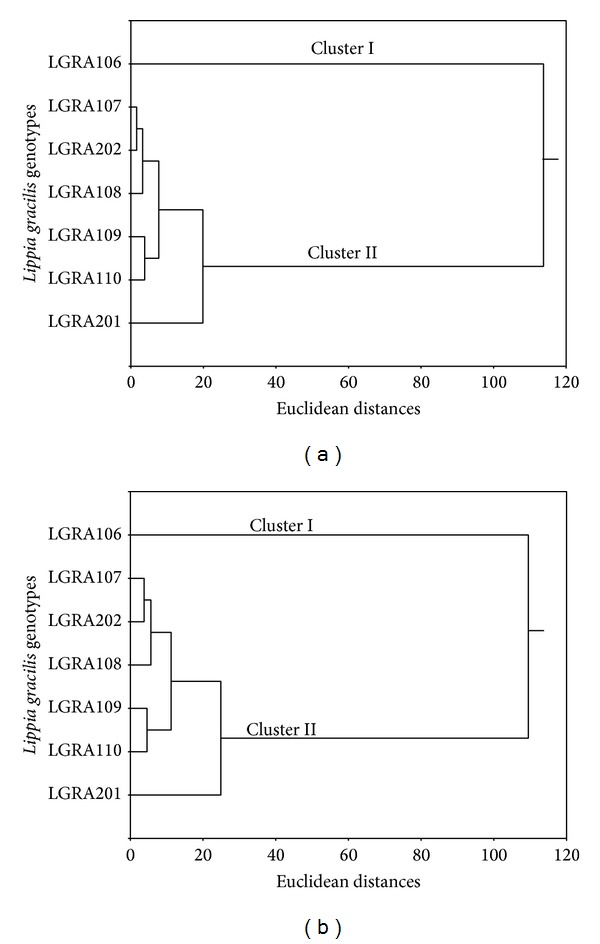
Bidimensional dendrograms representing the similarity of the chemical composition between seven* L. gracilis* genotypes for plants cultivated with (a) and without (b) irrigation.

Two groups were classified and grouped by chemical composition using multivariate analysis and differentiated by cluster analysis for the treatments with and without irrigation (Figures 4(a) and 4(b)).

**Figure 5:**
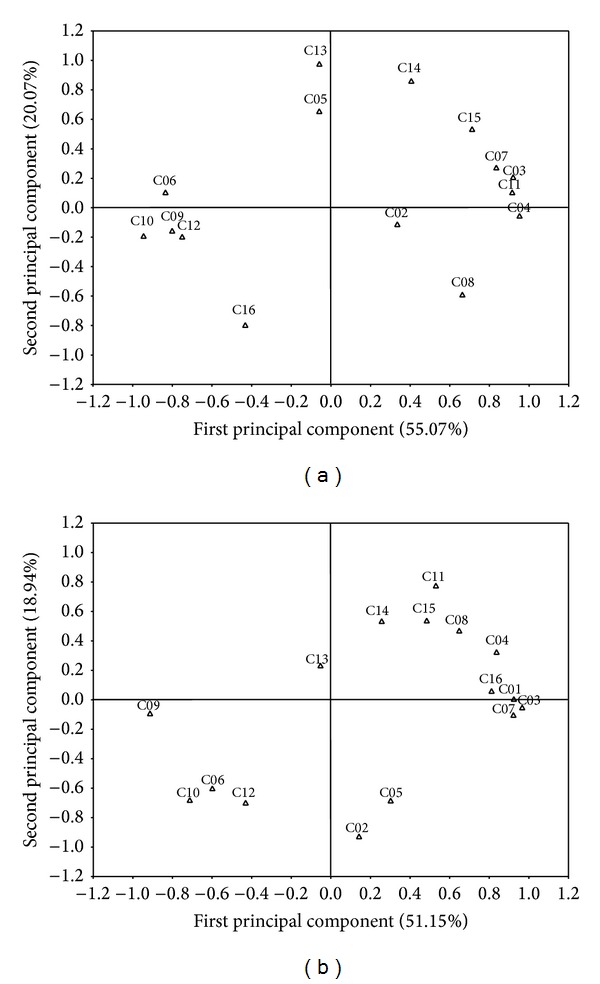
Distribution of the chemical constituents of the essential oil of* L. gracilis* in relation to the two principal components through analysis of the principal component analysis (PCA) for plants cultivated with (a) and without (b) irrigation. (C01 = α-thujene, C02 = myrcene, C03 = α-terpinene, C04 = p-cymene, C05 = limonene, C06 = 1,8-cineol, C07 = *γ*-terpinene, C08 = linalool, C09 = methyl thymol, C10 = thymol, C11 = carvacrol, C12 = *β*-caryophyllene, C13 = α-humulene, C14 = bicyclogermacrene, C15 = spathulenol, and C16 = caryophyllene oxide).

According to the analysis of the two principal components (Figures 5(a) and 5(b)), the first principal component of the treatment, with irrigation representing 55.07% of the variance, is positively related to α-thujene (*r* = 0.92), α-terpinene (*r* = 0.92), p-cymene (*r* = 0.95), *γ*-terpinene (*r* = 0.83), and carvacrol (*r* = 0.91) and negatively to 1,8-cineol (*r* = −0.84) and thymol (*r* = −0.95). The first principal component of the treatment without irrigation representing 51.15% of the variance, is positively related to α-thujene (*r* = 0.92), α-terpinene (*r* = 0.97), p-cymene (*r* = 0.84), and *γ*-terpinene (*r* = 0.92) and negatively to methyl thymol (*r* = −0.91) and thymol (*r* = −0.71).

The second principal component related positively to α-humulene (*r* = 0.97) and bicyclogermacrene (*r* = 0.86) and negatively to caryophyllene oxide (*r* = −0.80), in the treatment with irrigation. In the treatment without irrigation the second principal component related positively to carvacrol (*r* = 0.78) and negatively to myrcene (*r* = −0.93) (Figures 5(a) and 5(b)).

These statistical studies have established the chemical correlation between different genotypes, which is essential in the chemotaxonomic classification of aromatic plants [22, 23].

## 4. Conclusions

*L. gracilis* can be cultivated with or without irrigation and harvested in the rainy and dry season, maintaining the stability of the chemical composition of its essential oil.

In the Active Germplasm Bank of the Federal University of Sergipe there are two chemotypes defined, which are thymol and carvacrol, regardless of harvest season and irrigation.

The essential oil of* L. gracilis* genotype LGRA106 shows thymol as major compound and the genotypes LGRA107, LGRA108, LGRA109, LGRA110, LGRA201, and LGRA202 present carvacrol as major constituent.

## Figures and Tables

**Table 1:** Genotypes of *L. gracilis* present in the Active Germplasm Bank of medicinal plants of the Federal University of Sergipe.

Code	Origin	Geographical data	Voucher number
LGRA106	Tomar do Geru, Sergipe Sate, Brazil	11 19′ 16,7′′ S; 37 55′ 09,2′′ W	14733
LGRA107	Tomar do Geru, Sergipe Sate, Brazil	11 19′ 20,1′′ S; 37 55′ 13,5′′ W	14737
LGRA108	Tomar do Geru, Sergipe Sate, Brazil	11 19′ 22,4′′ S; 37 55′ 12,6′′ W	14734
LGRA109	Tomar do Geru, Sergipe Sate, Brazil	11 19′ 20,7′′ S; 37 55′ 16,9′′ W	14735
LGRA110	Tomar do Geru, Sergipe Sate, Brazil	11 19′ 21,1′′ S; 37 55′ 14,9′′ W	14732
LGRA201	Rio Real, Bahia State, Brazil	11 23′ 38,7′′ S; 38 00′ 54,1′′ W	14736
LGRA202	Rio Real, Bahia State, Brazil	11 23′ 45,3′′ S; 38 00′ 51,3′′ W	14731

**Table 2:** Chemical composition (%) of the essential oil of *L. gracilis* genotypes harvested in rainy and dry season.

Compound	RRI	*L. gracilis* genotypes						
		LGRA106	LGRA107	LGRA108	LGRA109	LGRA110	LGRA201	LGRA202
		Rainy season						
*α*-Thujene	924	0.45^bA^	0.98^bA^	0.98^bA^	0.85^bA^	0.95^bA^	1.37^aA^	1.00^bA^
Myrcene	976	2.74^bA^	2.65^bA^	2.05^cA^	2.06^cA^	3.10^aA^	3.20^aA^	3.02^aA^
*α*-Terpinene	988	1.00^cA^	2.28^bA^	1.79^cA^	1.82^cA^	2.27^bA^	3.01^aA^	2.23^bA^
p-Cymene	1016	6.70^cA^	11.46^bA^	11.75^bA^	13.02^aA^	12.87^aA^	13.74^aA^	13.30^aA^
Limonene	1023	0.36^aA^	0.38^aA^	0.38^aA^	0.21^bA^	0.37^aA^	0.44^aA^	0.41^aA^
1,8-Cineol	1028	3.92^aB^	0.61^dA^	2.10^bB^	0.0^dA^	2.50^bA^	1.50^cA^	0.72^dA^
*γ*-Terpinene	1031	3.66^eA^	13.52^bA^	8.81^dA^	8.55^dA^	11.81^cA^	21.11^aA^	11.99^cA^
Linalool	1057	0.41^bA^	0.80^aA^	0.44^bA^	0.79^aA^	0.54^bA^	0.72^aA^	0.73^aA^
Methyl thymol	1180	8.32^aB^	4.28^dB^	5.85^bB^	4.77^cB^	4.35^dB^	0.19^eA^	5.05^cA^
Thymol	1195	59.26^aB^	4.50^bA^	3.65^bA^	3.20^bA^	4.06^bA^	5.78^bA^	4.03^bA^
Carvacrol	1228	0.88^dA^	43.24^bA^	47.10^aA^	48.99^aA^	48.91^aA^	35.28^cA^	47.29^aA^
*β*-Caryophyllene	1291	8.57^aA^	6.20^bA^	3.92^aA^	7.80^aA^	4.44^aA^	6.26^bA^	3.86^aA^
*α*-Humulene	1298	0.47^cA^	0.85^bA^	1.00^aA^	0.38^dA^	0.33^dA^	0.49^cA^	0.29^dA^
Bicyclogermacrene	1432	0.07^cA^	1.40^bA^	1.95^aA^	0.55^cA^	0.44^cA^	1.05^bA^	0.42^cA^
Spathulenol	1437	0.19^dA^	0.62^bA^	1.36^aA^	0.58^bA^	0.33^dA^	0.69^bA^	0.48^bA^
Caryophyllene oxide	1454	0.74^aB^	0.58^bA^	0.62^bA^	0.71^aA^	0.56^bA^	0.82^aA^	0.57^bA^

Monoterpenes		87.70	84.70	84.90	84.26	91.73	86.34	89.77
Sesquiterpenes		10.04	9.65	8.85	10.02	6.10	9.31	5.62
Essential oil content (%)		1.25^aA^	1.70^aA^	1.60^aB^	1.35^aB^	1.52^aB^	1.92^aA^	1.52^aB^
Essential oil yield (mL*·*plant^−1^)		1.67^aA^	2.27^aA^	2.13^aB^	1.80^aB^	2.03^aB^	2.57^aA^	2.03^aB^

		Dry season						
*α*-Thujene	924	0.51^cA^	1.05^bA^	0.98^bA^	0.93^bA^	1.05^bA^	1.24^aA^	1.14^aA^
Myrcene	976	2.25^cB^	2.46^bA^	1.80^eB^	2.00^dA^	2.67^aB^	2.74^aB^	2.51^bB^
*α*-Terpinene	988	0.91^dA^	2.39^bA^	1.89^cA^	2.08^cA^	2.27^bA^	3.02^aA^	2.30^bA^
p-Cymene	1016	8.12^bA^	12.86^aA^	12.51^aA^	13.34^aA^	14.03^aA^	13.22^aA^	12.80^aA^
Limonene	1023	0.38^aA^	0.36^aA^	0.46^aA^	0.21^bA^	0.37^aA^	0.48^aA^	0.39^aA^
1,8-Cineol	1028	5.03^aA^	0.52^dA^	3.03^bA^	0.0^dA^	3.13^bA^	2.02^cA^	0.33^dA^
*γ*-Terpinene	1031	3.50^dA^	13.66^bA^	9.86^cA^	10.52^cA^	11.33^cA^	19.65^aA^	12.53^bA^
Linalool	1057	0.36^bA^	0.55^aA^	0.23^bA^	0.59^aA^	0.48^aA^	0.53^aA^	0.59^aA^
Methyl thymol	1180	10.78^aA^	5.40^bA^	6.97^bA^	5.93^bA^	6.03^bA^	0.23^cA^	6.01^bA^
Thymol	1195	56.77^aA^	4.42^cA^	3.25^dA^	3.50^dA^	3.76^dA^	6.95^bA^	4.53^cA^
Carvacrol	1228	0.64^dA^	43.74^bA^	44.00^bB^	48.29^aA^	45.40^bA^	36.57^cA^	44.41^bB^
*β*-Caryophyllene	1291	7.27^aA^	4.72^bB^	3.75^cA^	5.23^bB^	3.33^cA^	5.30^bA^	4.18^cA^
*α*-Humulene	1298	0.37^bA^	0.58^bA^	0.99^aA^	0.22^bA^	0.25^bA^	0.44^bA^	0.41^bA^
Bicyclogermacrene	1432	0.0^cA^	1.28^bA^	2.22^aA^	0.48^cA^	0.37^cA^	1.14^bA^	1.08^bA^
Spathulenol	1437	0.0^cA^	0.61^bA^	1.09^aA^	0.61^bA^	0.37^cA^	0.56^bA^	0.60^bA^
Caryophyllene oxide	1454	1.13^aA^	0.73^cA^	0.69^cA^	1.09^aA^	0.87^bA^	0.93^bA^	0.77^cA^

Monoterpenes		89,25	87,41	84,98	87,39	90,52	86,65	87,54
Sesquiterpenes		8,77	7,92	8,74	7,63	5,19	8,37	7,04
Essential oil content (%)		1.42^bA^	2.02^aA^	2.17^aA^	1.85^bA^	2.15^aA^	2.37^aA^	2.70^aA^
Essential oil yield (mL*·*plant^−1^)		1.90^bA^	2.70^aA^	2.90^aA^	2.47^bA^	2.87^aA^	3.17^aA^	3.60^aA^

RRI: relative retention index. Different lowercase letters indicate differences within lines (genotypes), and uppercase letters indicate differences between harvest seasons. Values followed by the same letter are not statistically different based on the Scott-Knott test (*P* ≤ 0.05).

**Table 3:** Chemical composition (%) of the essential oil of *L. gracilis* genotypes cultivated with and without irrigation.

Compound	RRI	*L. gracilis* genotypes						
		LGRA106	LGRA107	LGRA108	LGRA109	LGRA110	LGRA201	LGRA202
		With irrigation						
*α*-Thujene	924	0.51^cB^	1.05^bA^	0.97^bA^	0.93^bA^	1.00^bA^	1.24^aA^	1.25^aA^
Myrcene	976	2.25^bB^	2.46^aA^	2.00^bA^	2.00^bA^	2.63^aA^	2.74^aA^	2.40^aA^
*α*-Terpinene	988	0.91^cA^	2.39^bA^	2.12^bA^	2.08^bA^	2.24^bA^	3.02^aA^	2.30^bA^
p-Cymene	1016	8.12^bA^	12.86^aA^	12.08^aA^	13.34^aA^	14.28^aA^	13.22^aA^	12.80^aA^
Limonene	1023	0.38^bA^	0.36^bA^	0.42^bA^	0.21^cA^	0.36^bA^	0.48^aA^	0.39^bA^
1,8-Cineol	1028	5.03^aA^	0.52^cA^	2.27^bA^	0.00^cA^	2.65^bA^	2.02^bA^	0.33^cA^
*γ*-Terpinene	1031	3.50^cA^	13.66^bA^	11.54^bA^	10.52^bA^	11.45^bA^	19.55^aA^	12.53^bA^
Linalool	1057	0.36^bA^	0.55^aB^	0.28^bA^	0.59^aA^	0.45^aA^	0.56^aA^	0.59^aA^
Methyl thymol	1180	10.78^aA^	5.40^bA^	6.18^bA^	5.70^bA^	5.93^bA^	0.23^cA^	6.01^bA^
Thymol	1195	56.77^aA^	4.42^cA^	3.89^cA^	3.50^cA^	3.83^cA^	6.95^bA^	4.53^cA^
Carvacrol	1228	0.64^dA^	43.84^bA^	43.85^bA^	48.29^aB^	46.91^aA^	36.57^cA^	44.41^bA^
*β*-Caryophyllene	1291	7.27^aB^	4.72^bB^	4.22^cA^	4.96^bB^	3.21^cA^	5.30^bB^	4.18^cA^
*α*-Humulene	1298	0.37^cA^	0.58^bB^	0.88^aA^	0.16^dB^	0.24^dA^	0.44^cA^	0.44^cA^
Bicyclogermacrene	1432	0.00^bA^	1.28^bA^	2.03^aB^	0.48^bA^	0.24^bA^	1.14^aA^	1.08^aA^
Spathulenol	1437	0.00^cA^	0.61^aA^	0.84^aB^	0.61^aA^	0.35^bA^	0.56^aB^	0.60^aB^
Caryophyllene oxide	1454	1.13^aA^	0.73^bA^	0.62^bA^	1.09^aA^	0.84^bA^	0.93^aA^	0.77^bA^

Monoterpenes		89.25	87.51	85.60	87.16	91.73	86.58	87.54
Sesquiterpenes		8.77	7.92	8.59	7.30	4.88	8.37	7.07
Essential oil content (%)		1.42^cA^	2.02^bA^	2.17^bA^	1.85^bA^	2.15^bA^	2.87^aA^	2.77^aA^
Essential oil yield (mL*·*plant^−1^)		1.90^cA^	2.70^bA^	2.90^bA^	2.46^bA^	2.86^bA^	3.83^aA^	3.70^aA^

		Without irrigation						
*α*-Thujene	924	0.74^bA^	0.97^bA^	1.13^aA^	0.88^bA^	1.01^bA^	1.26^aA^	1.28^aA^
Myrcene	976	2.72^aA^	2.13^bB^	1.81^cA^	1.63^cB^	2.46^aA^	2.64^aA^	2.53^aA^
*α*-Terpinene	988	1.06^dA^	2.08^bB^	1.88^cA^	1.67^cB^	2.09^bA^	3.01^aA^	2.12^bA^
p-Cymene	1016	7.34^bA^	8.95^bB^	11.22^aA^	10.99^aB^	10.78^aB^	11.79^aA^	11.85^aA^
Limonene	1023	0.34^aA^	0.26^bA^	0.34^aA^	0.22^bA^	0.29^aA^	0.37^aB^	0.32^aA^
1,8-Cineol	1028	3.25^aB^	0.39^dA^	1.70^bA^	0.00^dA^	0.95^cB^	0.96^cB^	0.19^dA^
*γ*-Terpinene	1031	4.21^dA^	13.08^bA^	9.60^cB^	8.49^cB^	11.31^bA^	20.20^aA^	11.38^bA^
Linalool	1057	0.13^cB^	0.78^aA^	0.40^bA^	0.66^aA^	0.44^bA^	0.63^aA^	0.71^aA^
Methyl thymol	1180	10.08^aA^	4.07^cB^	6.24^bA^	5.00^cA^	4.40^cB^	0.17^dA^	5.07^cA^
Thymol	1195	53.62^aB^	5.03^bA^	3.80^bA^	3.72^bA^	4.82^bA^	6.23^bA^	4.61^bA^
Carvacrol	1228	0.90^dA^	45.96^bA^	44.98^bA^	51.35^aA^	52.24^aA^	35.16^cA^	46.12^bA^
*β*-Caryophyllene	1291	11.98^aA^	6.29^cA^	3.87^dA^	6.81^cA^	3.94^dA^	8.24^bA^	5.04^dA^
*α*-Humulene	1298	0.61^bA^	0.89^aA^	1.08^aA^	0.44^cA^	0.31^cA^	0.67^bA^	0.40^cA^
Bicyclogermacrene	1432	0.00^cA^	1.46^bA^	3.41^aA^	0.91^bA^	0.17^cA^	1.30^bA^	0.90^bA^
Spathulenol	1437	0.00^dA^	0.72^bA^	1.69^aA^	0.70^bA^	0.34^cA^	0.94^bA^	0.84^bA^
Caryophyllene oxide	1454	0.45^bB^	0.51^bB^	0.61^bA^	0.75^aB^	0.57^bB^	0.80^aA^	0.85^aA^

Monoterpenes		84.39	83.70	83.10	84.61	90.79	82.42	86.18
Sesquiterpenes		13.04	9.87	10.66	9.61	5.33	11.95	8.03
Essential oil content (%)		1.60^cA^	2.32^bA^	2.25^bA^	2.00^bA^	1.95^cA^	3.00^aA^	2.45^bA^
Essential oil yield (mL*·*plant^−1^)		2.01^cA^	3.10^bA^	3.00^bA^	2.66^cA^	2.60^cA^	4.00^aA^	3.20^bA^

RRI: relative retention index. Different lowercase letters indicate differences within lines (genotypes), and uppercase letters indicate differences between treatments with and without irrigation. Values followed by the same letter are not statistically different based on the Scott-Knott test (*P* ≤ 0.05).
